# Is the Concept of Delirious Mania Valid in the Elderly? A Case Report and a Review of the Literature

**DOI:** 10.1155/2013/432568

**Published:** 2013-07-28

**Authors:** Pramudith M. Maldeniya, Akshya Vasudev

**Affiliations:** Department of Psychiatry, Western University, London Health Sciences Centre, 800 Commissioners Road East, London, ON, Canada N6A 5W9

## Abstract

Delirious mania has been well recognized in the published literature and in the clinic. Over the years there has been refinement of understanding of its clinical features, course, and treatment. The literature suggests that delirious mania should be considered in individuals who present with a constellation of sudden onset delirium, mania, and psychosis. However, delirious mania is not recognized under a formal classification system nor are there any formal guidelines for its treatment. We, as such, question if the concept of delirious mania in the elderly is valid. We present a case of an elderly man with marked features of delirium with minimal manic or psychotic features who had a previous diagnosis of bipolar I disorder. On thorough clinical assessments no identifiable cause of his delirium was found. We therefore considered his presentation to be more likely due to delirious mania. Electroconvulsive therapy was considered and offered to which he responded very well. We invite the reader to consider whether delirious mania is a valid concept in the elderly, where features of delirium may be more prominent than manic or psychotic features.

## 1. Introduction

Delirious mania has been described as a clinical syndrome since 1832 by Calmeil [1]. The literature has recognized this concept in the form of clinical reports, but delirious mania has not been formally captured in a classification system such as the *Diagnostic and Statistical Manual of Mental Disorders *[2]. A reasonable definition of delirious mania is concurrence of rapid onset delirium, mania, and psychosis without association with a physical illness, prior toxicity, or another mental disorder [3]. Bell in 1849 identified that out of 1700 patients seen in McLean Asylum, USA, 40 patients presented with a “disease resembling some advanced stages of mania, and fever” [4]. He described features including a sudden onset of clouding of consciousness, delusions, hallucinations, overactivity, and sleeplessness. 

The term delirious mania was first coined by Kraepelin [5] in 1921. Kraepelin had categorized mania into three subtypes: delirious mania, acute mania and hypomania. He felt that there was a continuum from the mildest form, hypomania, to acute mania and finally delirious mania as the most severe form of mania [5]. Other definitions include those of Klerman who tried to expand the characterization of mania into 5 stages: normal, neurotic, hypomanic, manic, and delirious stages [6]. Carson and Goodwin [7] noted that six patients out of a series of twenty manic cases, besides having manic symptoms, were also disoriented to place and time. They were thought to have a presentation similar to Kraepelin's concept of delirious mania. 

At present, despite a lack of clear consensus or guidelines for definition and characteristics, Fink's [8] definition for delirious mania would probably hold as the one most agreed upon. He defined delirious mania as a rapid onset syndrome resulting in delirium, psychosis, and mania. Taylor and Fink [9] further theorized that delirious mania in fact could be a form of catatonia based on presence of catatonic features. However, there is still no consensus whether delirious mania should be classified as a form of catatonia or a subtype of bipolar disorder or perhaps even a primary psychiatric (nonorganic) underlying cause of delirium [8–10].

 Recently, Jacobowski et al. [3] reviewed the pathophysiology, diagnosis, and treatment strategies for delirious mania. Notably, features of delirium such as fluctuating sensorium, altered consciousness, disorientation, and catatonic features were the hallmark features described. They also noted the occurrence of manic symptoms of grandiosity, insomnia, and increase in psychomotor activity. 

However, delirious mania in the elderly has been sparsely reported. To our knowledge, Weintraub and Lippman [11] published one of the few case reports of delirious mania in an older population. Their case report describes two cases of elderly patients who initially presented in a state of delirium and were additionally noted to have distinct manic features during the latter part of their inpatient stay. They recommended that differential diagnosis for someone who presents with features of confusion, disorientation, and perceptual changes should include delirious mania, particularly so in the elderly [11]. Another case report authored by Rosenzweig et al. [12] described a case of an elderly retired teacher with no previous psychiatric history who developed delirium and manic symptoms in close association with a cycle of carboplatin and paclitaxel chemotherapy. It is possible that in this case the delirious manic symptoms were organic in etiology related to the chemotherapy being provided. Lee [13] described a series of 5 cases of delirious mania and discussed them in context of clinical review of delirious mania but only 1 of the cases involved an elderly patient. Similarly, Karmacharya et al. [14] evaluated 16 cases of delirious mania to determine characteristics and treatment responses. Only one of the 16 was close to an elderly age, 64. 

We present a case of a gentleman who initially presented with features consistent with delirium. No identifiable causes of delirium were discovered; hence, we considered a diagnosis of delirious mania even in the absence of florid manic and psychotic features. The patient had an excellent response to a short course of electroconvulsive therapy. Based on the relative paucity of manic and psychotic features in his presentation, we invite the reader to question whether the classical description of delirious mania is a valid clinical concept in the elderly.

## 2. Case Presentation

Mr. P. B. is a 73-year-old retired accountant with a history of bipolar (Type 1) managed by a community Psychiatrist. He has been divorced from his wife for 2 years having separated earlier from her for several years. He has a son and daughter who are his powers of attorney. Mr. P. B. had a history of 2 manic episodes in the remote past. Both of these manic episodes occurred around 12 years ago and were characterized by irritability, excess energy, and spending. In 2006 he had an episode of confusion, which was deemed secondary to lithium toxicity and resolved on reduction of his lithium dosage. 

His psychiatric medications at this presentation were lamotrigine 100 mg morning and 200 mg nightly, lithium 600 mg nightly, and quetiapine 75 mg nightly with additional 25 mg nightly as needed. Mr. P. B.'s other medical conditions included hypertension, type 2 diabetes mellitus, hypothyroidism, erectile dysfunction, possible alcohol abuse, and coronary artery disease. He was recently prescribed propranolol for a possible essential tremor. His other medications included gabapentin, levothyroxine, perindopril, amlodipine, and furosemide.

He was initially admitted to the medical service at University Hospital, London, Ontario, on December 9, 2012, with symptoms of increasing confusion and tremors. He was admitted under the neurology team for presumed lithium toxicity, based on symptoms of tremor, rigidity, and confusion. His lithium was held. In addition amlodipine and furosemide diuretics were also held as they were thought to potentially aggravate lithium toxicity. A serum alcohol level and urine toxicology screen were found negative. In addition, amoxicillin was given empirically for a suspected urinary tract infection as he grew mixed bacteria on a urine culture. 

Geriatric Psychiatry Consultation Liaison service was then consulted. They ruled out lithium toxicity as serum lithium levels were considered to be subtoxic (0.75 mmol/L December 10). Lithium was therefore reinstated at a dose of 600 mg nightly to prevent an affective disorder relapse. Delirium was considered as the main diagnosis possibly due to sustained anticholinergic effects of the recently stopped furosemide or a prodrome of his underlying bipolar disorder. Consideration was also given for an underlying dementia syndrome. However, no collateral information was available at that time to confirm this. A Mini Mental Status Exam [15] conducted on December 11 revealed a score of 18/30. Mr. P. B. was unable to spell the word “WORLD” backwards. He was also unable to recall 3 objects or repeat a sentence. He refused to write a sentence or copy the intersecting pentagons. He was able to follow 2 out of 3 stage commands. 

He was subsequently transferred on December 18 to the psychiatric inpatient unit at Victoria Hospital, a large district hospital with 74 beds dedicated to inpatients for mental health care based at London, Ontario, which has 10 beds dedicated to geriatric psychiatry. The dose of lithium was lowered to 150 mg nightly to see if that would improve his delirium. However, Mr. P. B. remained floridly confused and occasionally wandered around the unit in varying states of undress. He was occasionally found to be disorganized with regards to his thoughts and behaviours. Sometimes disorganized behaviours occurred. He was often not oriented to place and time with fluctuating cognition noted, varying from day to day. 

On better days Mr. P. B. was able to answer questions with regard to orientation to place and person. In these lucid intervals the patient was observed for the most part to have normal speech and thought processes with only occasional racing thoughts noted. Sleeping patterns were noted to be irregular. Occasionally Mr. P. B. woke up at night and was noted to lie on the floor mumbling to himself, being extremely disoriented, and wandered on the unit appearing confused. 

 Further medical workup was performed for a treatable cause of his confusion. Metabolic abnormalities were ruled out on the basis of normal ranges of complete blood count and serum glucose as well as electrolytes including sodium, potassium calcium, magnesium, chloride, and phosphate. Levels of vitamin B12 and folate were also found to be within normal range. An electroencephalogram (EEG) was performed which did not reveal any seizure activity. Chest X ray, blood culture, urine culture, and Venereal Disease Research Laboratory (VDRL) testing were also normal. A lumbar puncture was considered to rule out any central nervous system infection, but this was deemed unnecessary by neurology, as on their examination they found no focal neurological or systemic signs. 

Substance withdrawal was also ruled out based on son's collateral information of lack of significant substance abuse and dependence history. Neuroleptic malignant syndrome and serotonin syndrome were considered but not supported as potential diagnoses as there was no pyrexia, autonomic instability, or marked rigidity.

On computerized tomography (CT) imaging of the brain, an incidental finding of an enlarged pituitary mass was noted. A follow-up magnetic resonance imaging (MRI) on 21 December confirmed a 1.5 cm pituitary microadenoma with no optic chiasm compression. Apart from this some chronic ischemic brain changes were noted. A full body CT was ordered to rule out any metastasis and this was negative. In addition, pituitary hormone values were within normal ranges, including AM cortisol, follicle stimulating hormone, luteinizing hormone, and prolactin. Mr. P. B. remained on a maintenance dose of levothyroxine (75 micrograms daily) for hypothyroidism. TSH values of 0.97 mIU/L on December 18 suggested that the dosage of thyroxine was adequate for compensation. Free T3 value of 5.1 pmol/L and free T4 value of 16 pmol/L also supported adequate compensation and hence hypothyroidism was not considered contributing to his delirious presentation. 

On December 28 lamotrigine dosing was decreased to 100 mg po morning and 150 mg po nightly to rule out if these were contributing to his delirium. In addition quetiapine was stopped on December 30 due to its deliriogenic potential and instead risperidone 0.25 mg was started on December 30 for management of agitation associated with his delirium. It was increased to 0.5 mg nightly on an as-required basis to improve sleep as well. A neurologist was reconsulted who suggested trying 100 mg levodopa/25 mg carbidopa tablets three times daily for his parkinsonism symptoms NYD of increased muscle tone and bradykinesia in his upper limbs bilaterally with minor cogwheeling. 

Ongoing collateral history obtained from family revealed that Mr. P. B. was relatively high functioning at home with only minor short-term memory issues, with no aphasia, agnosias or visuospatial difficulties, and hence a baseline dementing syndrome was also ruled out. The family also mentioned that Mr. P. B. had started propranolol on November 29, 2012, but had had since then himself discontinued this after noting confusion and attributing it to this medication. 

Despite a lack of obvious organic causes, delirious features were persistent even 4 weeks into Mr. P. B.'s inpatient stay. He remained severely disoriented, often agitated and disorganized at some times, drowsy, and with decreased level of consciousness at others necessitating his continued stay in the psychiatric intensive care unit. Taking into account Mr. P. B.'s ongoing confusion, psychotropics were slowly withdrawn. On January 9, lithium was discontinued and from this date lamotrigine was tapered down slowly, by 50 mg weekly, until it was stopped in 3 weeks, January 29. It was noted that occasionally during his lucid intervals, Mr. P. B. had racing thoughts. Other subtle hypomanic symptoms such as slight expansiveness and lability in mood were also observed sporadically four weeks into his admission. These subtle manifestations coupled with a past history of Bipolar 1 disorder made us consider the possibility of a hitherto undiagnosed delirious mania. A second opinion was sought and ECT treatment was deemed appropriate as a treatment option. 

Mr. P. B. received his first ECT (bifrontal pulse width 0.5 milliseconds, frequency 40 hertz, duration 6 seconds, current 800 mA, charge 176) on January 17, 2013, after his son provided consent on the patient's behalf as a valid substitute decision maker. Subsequently, bitemporal ECT treatments (pulse width 0.5 mseconds, frequency 50 hertz, current 800 mA, duration 6 seconds) were given after discussing with son that this modality of treatment would likely give a quicker response but perhaps increase his confusion. At this time valproate semisodium enteric-coated 250 mg at bedtime was reinstated for mania prophylaxis. 

Mr. P. B. was oriented to month and city 2 days after bitemporal ECT number 3 and regaining orientation to all 3 spheres after ECT number 4. By February 4, he was able to better recollect daily details of his ward stay such as meals and activities. He became more cooperative and forthcoming with less episodes of agitation and a more goal oriented thought process coming into prominence. For the first time in his stay, we were able to assess Mr. J. B. using the Montreal Cognitive Assessment (MoCA) tool (12) on which he scored 15/30 on February 7. Risperidone was discontinued on February 11 as it was no longer seen as necessary. He was discharged on February 12 after completing six sessions of ECT and with his willingness to be followed by us, a caseworker, and appropriate social supports for medication compliance. He also agreed to have 4 more outpatient ECT treatment appointments.

On an outpatient follow-up appointment on 21 February, Mr. P. B. felt that his mental health was continuing to improve. His irregular sleeping patterns were normalizing and he was able to sleep through the night. He mentioned about occasionally still experiencing mild racing thoughts but felt that this had improved since the ECT treatments. He was now able to watch television, surf the internet, and converse with family members without trouble concentrating. His MoCA was reattempted and he scored 24/30. No mood and perceptual abnormalities were noted on mental state examination. 

On March 8, the day of his last outpatient appointment with us, the patient was appropriately reactive in his mood, with an animated affect, well groomed, and with no mood or perceptual abnormalities. He was completely oriented to time, place, and person with relatively maintained insight and judgment. He was discharged back to his community psychiatrist for long-term follow-up. He was advised to continue on valproate medication and to finish the course of ECT treatment (2 sessions remaining) over the next couple of weeks.

## 3. Discussion

Mr. P. B. met some of the features of a patient with delirious mania as summarized by Jacobowski et al. [3] and those described by other authors [8, 14, 16, 17]. A rapid onset of disorientation, fluctuating sensorium, altered level of consciousness, and severe cognitive dysfunction were noted. However, the absence of frank manic or psychotic symptoms were the predominant features of his presentation.

We observed only one subtle hypomanic feature of racing thoughts compared to previous descriptions of florid manic symptoms including intense excitement, disorganized rambling speech, hypersexuality, and inappropriate sexual behaviour [3]. Additional features such as grandiosity, emotionality lability, and irritability were not observed. On certain days, Mr. P. B. subjectively reported racing thoughts while on others disorganized thought process was noted. Insomnia was noted briefly on certain days rather than most days. We found this to be consistent with the irregular sleep patterns of delirium rather than a biological symptom of mania. We also noticed that he had slight lability and expansiveness in his affect. These subtle hypomanic symptoms along with exhaustion of other differential diagnoses late in his stay helped us consider delirious mania as the underlying pathology. 

It was unclear whether Mr. P. B. was experiencing hallucinations and delusions, which are also described as clinical features of delirious mania in the published literature [8, 14, 16, 17]. In addition, no catatonic symptoms were observed [10].

We found P. B.,'s presentation of particular interest and a diagnostic challenge for several reasons. Firstly, J. B. was an elderly gentleman and we were particularly concerned with missing any medical causes of delirium. Psychiatric manifestations may occur in elderly delirious patients as mentioned by Weintraub and Lippmann [11]. Mr. P. B. also did not actively demonstrate marked characteristic manic behaviours or classical catatonic features. In addition he did not appear to respond to mood stabilizers and we tapered these off due to lack of effect as well as the possibility of these being responsible for his ongoing delirium. He, however, had an excellent response to ECT, which is regarded as a definitive treatment for delirious mania [14]. 

Mr. P. B. has had a prior history of bipolar disorder with remote history of manic episodes. As Weintraub and Lippmann [11] had concluded with their case studies of elderly patients with delirious mania, it bears merit that this should be on the differential diagnosis of an elderly patient who presents initially with delirious presentation and a bipolar history. As they have pointed out, medically ill elderly patients are at risk of developing delirium, which can then yield neuropsychiatric type symptoms along with the delirious features [11]. Teasing out manic type symptoms as distinct from the delirious presentation may prove a challenge. Using a history of bipolar disorder to hold a relatively low threshold of suspicion for delirious mania might prove valuable.

Delirious mania has been considered to have an association with catatonia as well [9]. Jacobowski et al. [3] have pointed out two groups of catatonic symptoms, which have been noted in the literature: firstly, a cluster of excited symptoms such as excessive purposeless activity, aggression, violence, undressing of clothes, stereotypic behaviours, excitement, and increased purposeless activity [3, 18, 19]. The inhibited/stuporous cluster of catatonic symptoms has also been seen such as mutism, negativism, staring, catalepsy, inactivity, motor rigidity, and bizarre posturing [3, 18, 19]. Both of these forms of catatonic mania are noted to be associated with delirious mania [8, 16].

Mr. P. B. did not demonstrate the above-mentioned positive or negative classical catatonic signs. He did, however, arguably demonstrate more subtle catatonic features including severe disorganization with agitated behaviours such as undressing. He also had transient Parkinsonian features for which he was given a trial of carbidopa-levodopa briefly without any improvement. 

Jacobowski et al. [3] discuss a general treatment approach for delirious mania with “non malignant signs.” These involved the lack of catatonia, hypertension, tachycardia, fever, and muscular rigidity [3]. They noted that the use of mood stabilizers combined with benzodiazepines or ECT was noted to work with some populations. Mr. P. B. resolved with ECT and a low dose of valproate, which supports the use of this therapy as a definitive solution [14]. 

One possibility is that Mr. P. B.'s symptomatology revealed a delirious presentation and that the very subtle signs of mania we appreciated were merely a part of the presentation of delirium. We performed an extensive medical workup to rule out any other medical contributors to his delirium, and in addition there was an excellent response to ECT; hence, delirium as the sole diagnosis is unlikely. ECT has been found an effective therapy in the elderly for neuropsychiatric disorders such as catatonia, bipolar mania, schizophrenia, Parkinson's disease, and behavioural disturbance associated with dementia [20]. However, to the best of our knowledge ECT has not been reported as an effective treatment for delirium in the elderly or in other populations.

Another possibility would be that P. B. had presented with delirium, which had persisted, and that later on the discontinuation of his lithium led to an evolution of mania. The resolution of both delirious and manic symptoms with ECT, however, does not support mania evolution superimposed on protracted delirium.

Elderly patients are particularly prone to developing delirium and perhaps therefore amongst this population a diagnosis of delirious mania may be missed. We feel that obtaining a thorough psychiatric history and paying particular attention to bipolar disorder history may prove useful. Also, our case makes us question whether delirious mania is a valid concept in the elderly, particularly in situations where features of delirium may be more prominent than manic or psychotic features. The presentation may reflect delirium alone, which medically ill elderly are prone to, or delirium with later evolution of mania. Based on the atypicalness of Mr. P. B.'s presentation, we suggest that the classical features of mania and psychosis along with confusion are not essential for a diagnosis of delirious mania in the elderly population.
